# Processing of emotional connotations in Chinese monomorphic and compound words reflected by the early posterior negativity

**DOI:** 10.3389/fpsyg.2024.1426383

**Published:** 2024-08-09

**Authors:** Kai Zhang, Jiaxin Li, Feng Gu

**Affiliations:** ^1^School of Chinese Languages and Literatures, Lanzhou University, Lanzhou, China; ^2^Research Centre for Language, Cognition, and Neuroscience, Department of Chinese and Bilingual Studies, The Hong Kong Polytechnic University, Kowloon, Hong Kong SAR, China; ^3^Neurocognitive Laboratory for Linguistics and Semiotics, College of Literature and Journalism, Sichuan University, Chengdu, China; ^4^Digital Convergence Laboratory of Chinese Cultural Inheritance and Global Communication, Sichuan University, Chengdu, China

**Keywords:** emotion processing, Chinese words, early posterior negativity (EPN), event-related potentials (ERPs), affective neurolinguistics

## Abstract

Writing stands as one of humanity’s most profound inventions, facilitating the efficient sharing and transmission of vast amounts of information. Similar to images and facial expressions, visual (written) words possess the ability to evoke emotional connotations. Understanding how the brain perceives these emotional nuances encoded in highly symbolic visual words is a key focus of the emerging field of “affective neurolinguistics.” At the core of this inquiry lies the examination of the early posterior negativity (EPN), an event-related potentials (ERPs) component peaking around 300 ms after stimulus onset in the occipitotemporal scalp region. EPN has consistently emerged in response to emotional stimuli, encompassing pictures, faces, and visual words. However, prior research has notably lacked observation of EPN in response to Chinese emotional words, raising questions about potential differences in emotional processing between Chinese and other languages. Given the logographic nature of the Chinese writing system and the prevalence of compound words in the Chinese lexicon, this study aims to explore whether the emotional processing of Chinese monomorphic and compound words elicits an EPN response. Two experiments were conducted: Experiment 1 utilized one-character words (monomorphic words), while Experiment 2 employed two-character words (compound words). Participants were assigned a go/no-go task, instructed to respond to unknown words (word recognition task) or blue stimuli (color decision task). Data analysis using a data-driven mass univariate approach revealed significant ERP differences between emotional and neutral words. Notably, the time course, scalp topography, and cortical generators of the difference ERP presented a characteristic EPN response in both experiments. These findings strongly support the notion that the processing of emotional connotations in both Chinese monomorphic and compound words is reflected by the EPN, paving the way for future research using EPN as an emotion-related ERP component for investigating emotional processing of Chinese words.

## 1 Introduction

Emotions are fundamental to our daily interactions (Oatley and Duncan, 1994), allowing us to interpret and respond to signals that may evoke fear, harm, or joy. In the context of human evolution, emotions likely played a pivotal role; for instance, the ability to discern fear from predators and react swiftly is crucial for survival. This evolutionary pressure contributed to the development of our ability to discern emotions from various stimuli, including pictures, vocal tones, and facial expressions. Remarkably, despite visual words being highly symbolic and a relatively recent development in human evolutionary history, we can discern emotions from them much like we do from pictures and facial expressions (Eimer and Holmes, 2007; Luo et al., 2010; Bayer and Schacht, 2014). Moreover, in contemporary society, visual words have become the predominant medium for human communication. Therefore, understanding the neural mechanisms involved in perceiving emotions from visual words is crucial. This has led to the emergence of a new research field known as “affective neurolinguistics,” which seeks to integrate the study of emotion with language processing in the human brain (Hinojosa et al., 2020; Kissler, 2020; Hinojosa et al., 2023).

Event-related potentials (ERPs) have demonstrated to be an effective tool for investigating the neural mechanisms underlying both human language processing and emotion processing. Emotion processing is a complex and multifaceted process that includes the rapid evaluation of emotional stimuli, the generation and regulation of emotional responses, and the interaction between emotion and cognition (for reviews see Hamann and Canli, 2004; Pessoa and Adolphs, 2010; Etkin et al., 2011). This process relies on the coordinated work of multiple brain regions, particularly the prefrontal cortex, amygdala, and hippocampus (for reviews see Phelps and LeDoux, 2005; Sergerie et al., 2008; Palomero-Gallagher and Amunts, 2022). Just as some critical aspects of language processing are reflected by specific language-related ERP components, such as the N400 associated with the retrieval of semantic memory (Lau et al., 2008; Kutas and Federmeier, 2011) and the P600 linked to syntactic processing or semantic reintegration (Friederici, 2004; Kuperberg, 2007; Delogu et al., 2019; Aurnhammer et al., 2023), two emotion-related ERP components have been identified: the early posterior negativity (EPN) and the late positive complex (LPC). The EPN is elicited by emotional stimuli compared to neutral stimuli, typically occurring around 300 ms after stimulus onset, and is most prominently observed in the occipitotemporal scalp area (e.g., Schacht and Sommer, 2009b; Fruhholz et al., 2011). The LPC is also elicited by emotional stimuli compared to neutral stimuli, but is prominently observed between 500 and 800 ms at centro-parietal electrodes (e.g., Cuthbert et al., 2000; Kissler et al., 2009). The EPN reflects the early cortical responses to emotional stimuli (Kissler et al., 2007; Schacht and Sommer, 2009b; Bayer et al., 2012a), whereas the LPC reflects more detailed or prolonged processing of emotional characteristics (Schacht and Sommer, 2009b; Bayer and Schacht, 2014; Zhang et al., 2014).

As a significant emotion-related ERP component, the EPN has been widely utilized in various studies investigating the neural mechanisms across different facets of emotional processing. These studies include: (1) Examining how the brain reacts to different attributes of emotional words, including word size (Bayer et al., 2012a), word class (nouns, verbs, and adjectives) (Herbert et al., 2008; Schacht and Sommer, 2009b; Palazova et al., 2011), word type (emotion-label or emotion-laden words) (Wu et al., 2021), and word concreteness (Palazova et al., 2013; Xu et al., 2017, 2019). (2) Investigating how attention and task demands modulate emotional processing (Schacht and Sommer, 2009b; Fruhholz et al., 2011; Gonzalez-Villar et al., 2014; Hinojosa et al., 2014; Delaney-Busch et al., 2016). (3) Examining the different role of valence and arousal in emotional processing (Leite et al., 2012; Citron et al., 2013; Espuny et al., 2018). (4) Investigating how demographic factors influence emotional processing, such as age (Wieser et al., 2006), gender (Rozenkrants and Polich, 2008; Kim et al., 2013), and bilingualism (Chen et al., 2015; Kissler and Bromberek-Dyzman, 2020; Naranowicz et al., 2022; Cieślicka and Guerrero, 2023). (5) Exploring abnormal emotion processing in specific populations, such as epileptic patients (Ponz et al., 2014), alcohol-dependent patients (Dominguez-Centeno et al., 2020), smokers (Deweese et al., 2018; Cui et al., 2019), individuals with highly schizotypal traits (Hoid et al., 2020), and individuals with trait anxiety (Liu B. et al., 2018).

These diverse applications of the EPN have illuminated several potential neural mechanisms underlying its elicitation. EPN may reflect arousal-driven attention allocation to emotional stimuli (Kissler et al., 2009; Citron, 2012; Gao et al., 2023), enhanced sensory encoding of emotional stimuli (Schupp et al., 2004b; Schacht and Sommer, 2009a; Chen and Wei, 2023), or semantic accessing of emotional words (Kissler et al., 2007; Herbert, 2022). Although the precise neural mechanisms underlying the EPN remain elusive, it is recognized as a crucial emotion-related ERP component in emotion research. However, intriguingly, the EPN is rarely observed when Chinese words serve as stimuli. A meticulous review of the literature yielded 24 studies that compared ERPs between Chinese words of varying emotionality. Surprisingly, only four of these 24 studies observed the EPN response. Table 1 offers a comprehensive overview of these 24 studies, detailing the experimental tasks, stimuli, and offline reference electrodes used in acquiring the ERPs. Such findings raise questions regarding whether the emotional processing of Chinese words diverges from that of other languages.

**Table 1:** Summary of ERP studies on emotion processing of Chinese words.

Study	Task	Stimuli	Morphology of stimuli	Arousal values of stimuli	Offline reference	EPN
Liu et al. (2010a)	Emotion evaluation	20 positive, 20 negative, 20 neutral	Two-character words	Positive = negative > neutral	Mean mastoids	–
Liu et al. (2010b)	Emotion evaluation	32 positive, 32 negative, 32 neutral	Two-character words	Positive = negative > neutral	Mean mastoids	–
Schacht et al. (2012)	Word recognition	6 positive, 6 negative, 6 neutral	Two-character words	Positive = negative > neutral	Global average	–
Yang et al. (2013)	Word counting	16 highly (8 positive, 8 negative), 16 moderately (8 positive, 8 negative), 16 neutral	Two-character words	Positive = negative = neutral	Mean mastoids	–
Liu et al. (2014)	Emotion evaluation	20 positive, 20 negative, 20 neutral	Two-character words	Positive = negative = neutral	Mean mastoids	–
Wang and Bastiaansen (2014)	Color detection	42 positive, 42 negative, 42 neutral	Two-character words	Positive = negative > neutral	Mean mastoids	–
Yao and Wang (2014)	Lexical decision	40 abstract words (20 positive, 20 negative), 40 concrete words (20 positive, 20 negative)	Two-character words	Positive = negative	Mean mastoids	–
Zhang et al. (2014)	Odd-even decision, emotion evaluation	6 positive, 6 negative, 6 neutral	Two-character words	Positive = negative = neutral	Global average	+
Chen et al. (2015)	Lexical decision	60 positive, 60 negative, 60 neutral	Two-character words	Positive = negative > neutral	Global average	+
Wei et al. (2016)*	Color decision	96 negative, 96 neutral	Two-character words	Negative > neutral	Mean mastoids	–
Yao et al. (2016), Exp1	Lexical decision	40 high-arousal words (20 positive, 20 negative), 40 low-arousal words (20 positive, 20 negative), 20 concrete neutral words	Two-character words	High arousal > low arousal > neutral	Mean mastoids	–
Yao et al. (2016), Exp2	Lexical decision	40 high-arousal words (20 positive, 20 negative), 40 low-arousal words (20 positive, 20 negative), 20 abstract neutral words	Two-character words	High arousal > low arousal > neutral	Mean mastoids	–
Wang and Li (2017)	Priming-identify	10 “expression,” 10 “control,” 20 “unrelated”	Two-character words	Control = expression = unrelated	Mean mastoids	–
Xu et al. (2017)	Lexical decision	40 emotion nouns, 40 abstract nouns, 40 concrete nouns	Two-character words	Emotion = abstract = concrete	Mean mastoids	–
Liu X. et al. (2018)*	Color perception	20 low-arousal positive, 20 low-arousal neutral	Two-character words	Positive = neutral	Mean mastoids	–
Zhang et al. (2018)	Odd-even decision, emotion evaluation	34 negative, 34 ambiguous neutral, 34 unambiguous neutral	One-character words	Negative > neutral	Mean mastoids	–
Zhao et al. (2018)	Odd-even decision, emotion evaluation	6 positive, 6 negative, 6 neutral	Two-character words	Positive = negative = neutral	Mean mastoids	–
Wang et al. (2019)	Lexical decision	60 emotion-label words (30 positive, 30 negative), 60 emotion-laden words (30 positive, 30 negative), 60 neutral words	Two-character words	Positive = negative > neutral	Global average	–
Xu et al. (2019)	Lexical decision	48 emotion nouns, 48 abstract nouns, 48 concrete nouns	Two-character words	Emotion > abstract = concrete	Mean mastoids	–
Ku et al. (2020)	Lexical decision	108 positive, 108 negative, 108 neutral	Two-character words	Positive = negative > neutral	Mean mastoids	–
Gu et al. (2021)	Semantic categorization	60 disgust-related, 60 neutral	Two-character words	Disgust-related = neutral	Global average	+
Luo and Qi (2022)	Odd-even decision, emotion evaluation	18 concrete words (6 positive, 6 negative, 6 neutral), 18 abstract words (6 positive, 6 negative, 6 neutral)	Two-character words	Positive = negative = neutral	Global average	–
Jia et al. (2022)	Affective Simon, lexical decision	90 emotion-label (45 positive, 45 negative), 90 emotion-laden (45 positive, 45 negative), 60 neutral	Two-character words	Positive = negative > neutral	Mean mastoids	–
Gao et al. (2023)	Emotion evaluation	50 positive, 50 neutral, 50 negative	Two-character words	Positive = negative > neutral	Global average	–
Liu et al. (2023)	Emotional categorization, emotional Stroop	68 emotion-label words (34 positive, 34 negative), 68 emotion-laden words (34 positive, 34 negative)	Two-character words	Positive = negative	Global average	+

This table presents comprehensive information on the experimental tasks, stimuli, electrode re-referencing configurations, and the presence (denoted as “+”) or absence (denoted as “–”) of the EPN component in each study. *While Wei et al. (2016) and Liu X. et al. (2018) reported EPN in their articles, the observed EPN manifested as positive deflections to emotional stimuli relative to neutral stimuli in the occipitotemporal scalp area. This polarity reversal diverges from the typical EPN response, therefore precluding its classification as a genuine EPN response.

Chinese is a prominent global language, with approximately 16% of the world’s population using it as their first language^1^. Chinese words exhibit two distinctive characteristics that differentiate them from many other languages. Firstly, Chinese employs characters as the building blocks of words (Wang, 1973). These characters are logographic symbols designed to convey meaning rather than pronunciation. Each character, characterized by a square shape, is formed from a combination of diverse strokes (e.g., “马,” meaning “horse,” and “星,” meaning “star”). While each character usually represents a meaningful morpheme, its pronunciation often cannot be inferred directly from its visual form. Secondly, the lexicon of modern Chinese predominantly consists of compounds. Specifically, one-character words make up only 5.5% of common vocabulary, while two-character words account for 72.1%, and multi-character words make up the remaining 22.4% (Lexicon of Common Words in Contemporary Chinese, 2021). Consequently, the majority of Chinese words are composed of two or more characters. Given that each character typically representing a distinct morpheme, it is common for Chinese words to be compound in nature [e.g., “电脑,” meaning “computer,” is composed of two characters (or morphemes) “电” and “脑,” where the former means “electric” and the latter means “brain”].

Several factors may contribute to the rare observation of the EPN response in previous studies focusing on Chinese emotional word processing. Firstly, the unique logographic nature of the Chinese writing system might influence the emotional processing of visual words differently. As introduced, Chinese words consist of logographic symbols that convey meaning rather than pronunciation, which sets them apart from alphabetic words such as English words. However, existing research indicates similarities in orthographic, lexical, and semantic processing between Chinese and alphabetic words (Bolger et al., 2005; Liu et al., 2008; Lin et al., 2011; Rueckl et al., 2015; Zhang et al., 2023). Therefore, it is unlikely that the logographic writing system alone leads to the absence of the EPN response in Chinese emotional word processing. Secondly, a notable observation is that almost all the studies listed in Table 1 utilized Chinese two-character words as stimuli. As introduced, Chinese two-character words are inherently compound words, comprising two constituent morphemes. Such compound words are processed both as a whole unit (whole-word or full-list processing) and as a combination of their constituent morphemes (decomposing or full-parsing processing) (Schreuder and Baayen, 1995; Pollatsek et al., 2000; Semenza and Luzzatti, 2014). Importantly, ERP investigations indicate that whole-word and decomposing processing broadly influence the ERP amplitude spanning 100–400 ms (El Yagoubi et al., 2008; Lavric et al., 2012; Fiorentino et al., 2014; Wu et al., 2016; Schmidtke and Kuperman, 2019; Tsang and Zou, 2022; Huang et al., 2023; Wei et al., 2023; for a review see Schmidtke and Kuperman, 2019), which overlaps with the typical EPN peak around 300 ms. Consequently, when two-character words serve as stimuli, the EPN response may be confounded by the ongoing whole-word and decomposing processing. Lastly, the choice of offline reference can significantly influence the morphology of the EPN response. When using the global average reference (i.e., mean ERP amplitude across all recording electrodes), the EPN typically exhibits a positive deflection over the frontal scalp region and a negative deflection over the occipitotemporal scalp region (Kissler et al., 2007, 2009; Schacht and Sommer, 2009b; Bayer et al., 2012b; Chen et al., 2015). Utilizing mean mastoids as the reference could potentially diminish the negative deflection over the occipitotemporal area due to its proximity to the mastoids. Thus, the global average reference is more appropriate for capturing the EPN response (Olofsson et al., 2008; Delaney-Busch et al., 2016; Imbir et al., 2021; Gibbons et al., 2022). Notably, a majority of the studies listed in Table 1 opted for the mean mastoids reference over the global average reference.

Drawing upon the considerations outlined above, the current study aims to revisit whether Chinese emotional words can evoke the EPN response. To this end, we conducted two experiments. The first experiment (Experiment 1) employed one-character words as stimuli, while the second experiment (Experiment 2) utilized two-character words. The outcomes of these experiments are intended to elucidate whether the EPN is elicited by monomorphic and compound words, respectively. To determine the presence or absence of the EPN response in a rigorous manner, we employed the mass univariate approach (Groppe et al., 2011), a data-driven statistical technique that offers a conservative assessment without prior knowledge about the specific electrodes or timings of the EPN response (Luck and Gaspelin, 2017). Such an approach allowed us to investigate the manifestation of the EPN without being constrained by predefined expectations. Moreover, in each experiment, participants engaged in both a word recognition task and a color decision task. Given that the EPN response is generally unaffected by the nature of the experimental task (e.g., Franken et al., 2009; Kissler et al., 2009; Schacht and Sommer, 2009b; for a review see Citron, 2012), the results from these two tasks serve as a means to validate and corroborate our findings. Lastly, the global average reference was utilized in this study to detect the presence or absence of the EPN response, as this reference is particularly optimized for capturing the EPN response.

## 2 Materials and methods

### 2.1 Participants

Thirty-two native Chinese speakers participated in the study. Two participants were excluded due to extensive artifacts during EEG recording. The remaining 30 participants comprised 15 females and 15 males, with ages ranging from 19 to 28 years (Mean = 22.6, SD = 2.4). They were undergraduate or graduate students studying at Sichuan University. Additionally, all participants were right-handed, as assessed by the Edinburgh Inventory (Oldfield, 1971), and had normal or corrected-to-normal vision. None reported color blindness or any history of mental illness, and they also indicated no prior difficulties in reading or writing. Informed consent forms were obtained from all participants before the experiment, and payment was administered after completion. The experimental procedure received approval from the Ethics Committee of Sichuan University.

### 2.2 Stimuli

In Experiment 1, a total of 150 Chinese one-character words were used as stimuli, consisting of 50 positive, 50 negative, and 50 neutral words (see Supplementary Table S1). The three emotion categories were carefully matched for visual structure (e.g., left–right, up-down, and semi-encircled patterns), stroke numbers (serving as an index of the visual complexity of Chinese characters), and character frequency (refer to Table 2). These stimuli were evaluated by 20 college students (aged 18–29 years, 12 female and 8 male) who were not participants in the EEG experiment. Using a 9-point Likert scale (Bradley and Lang, 1994), the students assessed the valence, arousal, and concreteness of each word. On this scale, “1” represented extreme negativity, minimal arousal, or high abstraction, while “9” represented extreme positivity, maximal arousal, or high concreteness. The mean valence, arousal, and concreteness scores are detailed in Table 2. A one-way analysis of variance (ANOVA) was conducted using valence, arousal, and concreteness as between-item factors. Results revealed a significant main effect of valence [*F*(2, 147) = 1527.8, *p* < 0.0001], and *post hoc* pairwise comparisons indicated significant differences in valence between any two emotion categories (*P*s < 0.0001, Bonferroni corrected). The main effect of arousal was also significant [*F*(2, 147) = 165.0, *p* < 0.0001], with post-hoc pairwise comparisons revealing significant differences in arousal between positive and neutral words (*p* < 0.0001, Bonferroni corrected), and between negative and neutral words (*p* < 0.0001, Bonferroni corrected), but no significant difference between positive and negative words (*p* = 1.000). Lastly, the main effect of concreteness was not significant [*F*(2, 147) = 2.004, *p* = 0.139].

**Table 2:** Specifications of the one-character words used in Experiment 1.

	Word category					
Variables	Positive		Negative		Neutral	
	Mean	SD	Mean	SD	Mean	SD
Valence	6.95	0.47	2.78	0.40	5.05	0.21
Arousal	5.75	0.58	5.71	0.66	4.02	0.33
Concreteness	5.75	0.83	5.82	0.69	6.08	1.05
Stroke number	8.82	1.84	8.98	2.03	9.32	1.74
Character frequency	147.76	153.79	146.89	145.49	146.05	236.16

Valence, arousal, and concreteness values were assessed using a 9-point Likert scale (Bradley and Lang, 1994). Stroke number serves as an indicator of the visual complexity of Chinese characters. Character frequency values (unit: per million) were sourced from the SUBTLEX-CH dataset (Cai and Brysbaert, 2010).

In Experiment 2, a total of 150 Chinese two-character words were used as stimuli, consisting of 50 positive, 50 negative, and 50 neutral words (see Supplementary Table S2). The three emotion categories were meticulously matched for stroke numbers and word frequency (refer to Table 3). Another group of 20 college students (aged 20–28 years, 11 female and 9 male) evaluated the valence, arousal, and concreteness of each word using the same methodology as described in Experiment 1. The mean valence, arousal, and concreteness scores are detailed in Table 3. A one-way ANOVA was conducted using valence, arousal, and concreteness as between-item factors. Results revealed a significant main effect of valence [*F*(2, 147) = 1302.7, *p* < 0.0001], and *post hoc* pairwise comparisons indicated significant differences in valence between any two emotion categories (*P*s < 0.0001, Bonferroni corrected). The main effect of arousal was also significant [*F*(2, 147) = 180.3, *p* < 0.0001], with post-hoc pairwise comparisons revealing significant differences in arousal between positive and neutral words (*p* < 0.0001, Bonferroni corrected), and between negative and neutral words (*p* < 0.0001, Bonferroni corrected), but no significant difference between positive and negative words (*p* = 1.000). Lastly, the main effect of concreteness was not significant [*F*(2, 147) = 0.133, *p* = 0.875].

**Table 3:** Specifications of the two-character words used in Experiment 2.

	Word category					
Variables	Positive		Negative		Neutral	
	Mean	SD	Mean	SD	Mean	SD
Valence	7.00	0.51	2.86	0.38	5.22	0.30
Arousal	5.83	0.68	5.86	0.58	3.85	0.55
Concreteness	6.19	0.67	6.11	0.90	6.19	1.03
Stroke number	17.18	4.32	17.00	4.53	16.78	4.93
Word frequency	414.80	556.79	375.90	464.49	400.86	441.60

Valence, arousal, and concreteness values were assessed using a 9-point Likert scale (Bradley and Lang, 1994). Word frequency values (unit: per million) were sourced from the SUBTLEX-CH dataset (Cai and Brysbaert, 2010).

In both experiments, each emotion category included only 50 words, necessitating multiple repetitions of these words to obtain sufficient trials for averaging the ERPs. Previous studies have demonstrated that stimulus repetition does not significantly influence the EPN (e.g., Schupp et al., 2006; Kissler et al., 2007). Consequently, the use of repeated stimuli is common in EPN research (e.g., Flaisch et al., 2008; Herbert et al., 2008; Kissler et al., 2009; Bayer et al., 2012b; Gonzalez-Villar et al., 2014), as was the case in the present study.

### 2.3 Procedure

**Figure 1:**
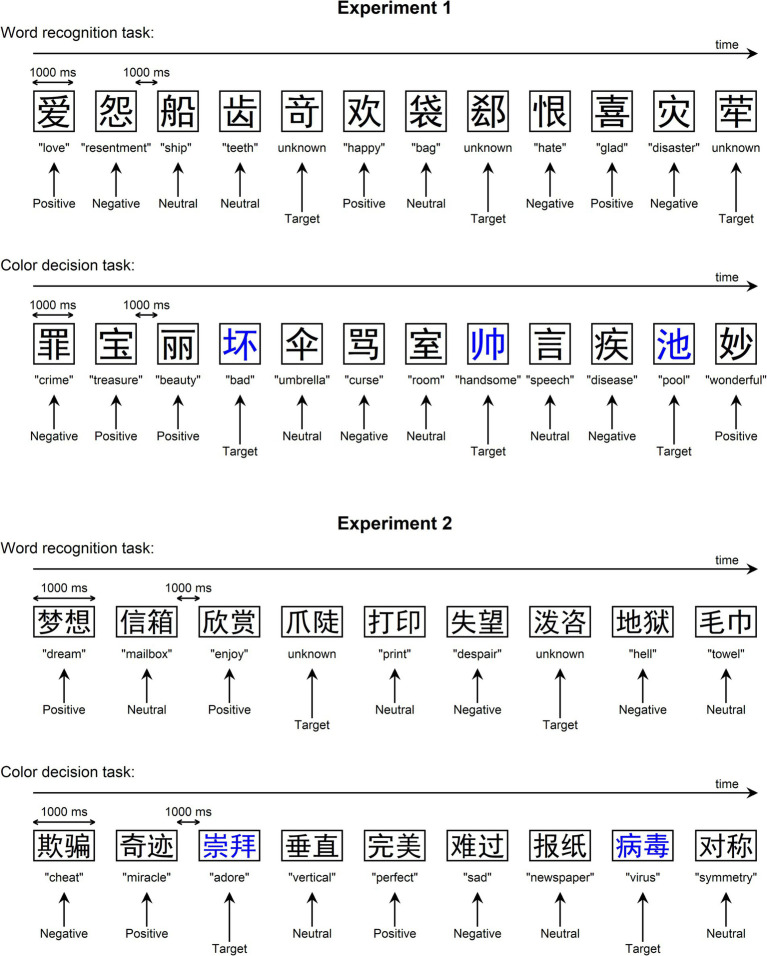
Experimental procedure. For both experiments, a go/no-go paradigm was employed for two tasks: a word recognition task and a color decision task. Participants were instructed to press a key button promptly upon encountering a target stimulus (i.e., an unknown word in the word recognition task and a blue stimulus in the color decision task). The English meaning of each Chinese word was provided.

Experiment 1 employed a go/no-go paradigm for two tasks: a word recognition task and a color decision task. During these tasks, stimuli were presented individually in black color at the center of a white background screen using the Heiti font. Each stimulus was displayed for 1,000 ms, followed by a 1,000 ms offset-to-onset interval (see Figure 1). Participants were seated in front of the monitor at a viewing distance of 140 cm. The size of each stimulus was 4.9 cm × 4.9 cm (equivalent to 2° × 2° in visual angle).

For the word recognition task, a total of 150 words (50 positive, 50 negative, and 50 neutral) were pseudorandomly presented with the following constraints: (1) consecutive appearances of the same word were avoided, and (2) words with the same emotional category (negative, positive, or neutral) were not repeated consecutively more than three times. Additionally, another set of 150 Chinese rare words (all were one-character words) served as targets (see Supplementary Table S1), matched to the 150 words in terms of visual structure and stroke number. These rare words had exceedingly low frequency, as they were not even available in the Chinese SUBTLEX-CH lexicon (Cai and Brysbaert, 2010). Hence, these rare words were labeled as “unknown words” because participants were unfamiliar with their orthography, pronunciation, and meaning. By utilizing these unknown words, a word recognition task was conducted. Participants were instructed to press a button using their index finger upon encountering an unknown word (i.e., target). Half of the participants used their left hands for button presses, while the other half used their right hands. The word recognition task comprised a total of 600 stimuli, encompassing 150 go trials (i.e., each unknown word appearing once) and 450 no-go trials (i.e., each known word appearing three times). The task was divided into two blocks, each lasting 10 min. Prior to the main task, participants underwent a brief practice session to acquaint themselves with the procedure. Notably, the go/no-go paradigm was employed for two reasons: firstly, it required participants to respond to only a few stimuli, reducing the inter-stimulus interval and the overall experiment duration, thus helping to prevent participant fatigue; secondly, it ensured that there was no influence of behavioral button presses on the ERPs to no-go trials.

The color decision task followed a similar procedure to the word recognition task, with the exception that the 150 unknown words in the go trials were substituted with the 150 one-character words (50 positive, 50 negative, and 50 neutral) presented in blue color. Participants were instructed to press a button promptly and accurately using their finger upon encountering a blue character (refer to Figure 1).

The order of the two tasks (two blocks for each task) was counterbalanced both within and between participants, with half of the participants assigned to WCCW and the other half to CWWC, where W stands for the word recognition task and C for the color decision task. After each block, participants were given a brief break. The experiment was conducted in a soundproofed room to minimize external distractions. The entire experimental session, including electrode application and removal, lasted approximately 1.5 h for each participant. Stimuli presentation and behavioral data recording were facilitated through E-Prime (ver 3.0).

The experimental procedure for Experiment 2 closely mirrored that of Experiment 1, with the exception that the stimuli consisted of two-character words (see Figure 1). Each word was displayed at a size of 9.8 cm × 4.9 cm, equivalent to 4° × 2° in visual angle. In Experiment 2, the “unknown words” (pseudowords) were generated by pairing two Chinese characters to form two-character pseudowords that do not exist in the Chinese SUBTLEX-CH lexicon (Cai and Brysbaert, 2010). The stroke numbers of the 150 pseudowords were matched with the 150 words (i.e., 50 positive, 50 negative, and 50 neutral). Half of the participants completed Experiment 1 prior to Experiment 2, whereas the remaining half started with Experiment 2. A gap of approximately 1 week separated the two experiments for each participant.

### 2.4 Electroencephalogram (EEG) recording

The EEG data was collected using a SynAmps 2 amplifier (NeuroScan, Charlotte, NC, United States) with 64 silver-chloride electrodes placed within an elastic cap on the scalp. The AC mode was used, capturing EEG signals within the frequency range of 0.05 to 400 Hz. The electrode placement followed the extended international 10/20 system. Additionally, electrical activity was recorded from both left and right mastoids. Vertical eye movements were monitored using a bipolar electrode (VEOG) above and below the left eye. The grounding electrode was located at AFz (between FPz and Fz). All recording electrodes were referenced to the tip of the nose, and electrode impedance was kept below 5 kΩ. We continuously recorded EEG data at a sampling rate of 500 Hz.

### 2.5 ERP analysis

For each experiment, the EEG data for each participant underwent a multi-step analysis process: (1) Ocular artifact correction: blink artifacts were corrected using a regression-based procedure. (2) Offline referencing: the EEG data were referenced to the global average (i.e., the mean voltage across all 64 electrodes). (3) Filtering: a digital bandpass filter (0.1–25 Hz) using a finite impulse response (FIR) filter was applied to isolate the desired brain activity range. (4) Epoch segmentation: the continuous data was segmented into 600-ms epochs, each containing a 100-ms pre-stimulus baseline period. (5) Baseline correction: the 100-ms pre-stimulus baseline was used to correct for any ongoing drifts in the EEG signal. (6) Artifact rejection: epochs with extreme voltage fluctuations exceeding ±75 μV in any channel, excluding the VEOG channel, were excluded. The remaining artifact-free epochs were then averaged for each condition. (7) ERP calculation by condition: ERPs were computed separately for emotional (positive and negative) and neutral stimuli within each task.

### 2.6 Statistical comparison of ERPs

For each experiment, ERPs elicited by emotional and neutral stimuli were statistically compared across all 64 scalp electrodes (excluding the VEOG channel). This involved repeated-measures analysis with two-tailed *t*-tests conducted at each time point from 50 to 500 ms. This resulted in a total of 14,400 comparisons. To address the issue of multiple comparisons and control for the false discovery rate (FDR), we employed the Benjamini-Hochberg procedure (Benjamini and Hochberg, 2018). The FDR represents the average proportion of statistically significant results that are likely false positives. In this study, the FDR was set at a level of 5%. The *t*-tests incorporating FDR control were performed using the Mass Univariate ERP Toolbox (Groppe et al., 2011).

### 2.7 Source analysis

For the source analysis of ERP, we employed the Surface Minimum Norm Image approach (Hämäläinen and Ilmoniemi, 1984) to identify the cortical generators. This was facilitated using the four-shell ellipsoidal head model in BESA Research (Version 7.1, BESA GmbH, Germany, http://www.besa.de/). The minimum norm approach is commonly used to compute a distributed electrical current image across the brain at each time sample.

## 3 Results

### 3.1 Behavioral results

Across all participants, the average accuracy in target identification was notably high (refer to Table 4), suggesting that participants closely followed the task instructions and executed the experiment with precision. In both experiments, paired-samples *t*-tests revealed that the average response time for the color decision task was significantly shorter than that for the word recognition task [Experiment 1: *t*(29) = 19.78, *p* < 0.0001, two-tailed; Experiment 2: *t*(29) = 21.28, *p* < 0.0001, two-tailed]. This difference reflected the reduced cognitive demands of the color decision task compared to the word recognition task.

**Table 4:** Behavioral results.

	Task	Mean accuracy (%)	Mean response time (ms)
Experiment 1	Word recognition	96.63 (SD = 3.81)	558.59 (SD = 54.30)
	Color decision	99.62 (SD = 1.09)	421.12 (SD = 55.68)
Experiment 2	Word recognition	98.17 (SD = 1.90)	596.95 (SD = 57.31)
	Color decision	99.71 (SD = 0.52)	426.85 (SD = 64.93)

### 3.2 ERP differences between emotional and neutral words

**Figure 2:**
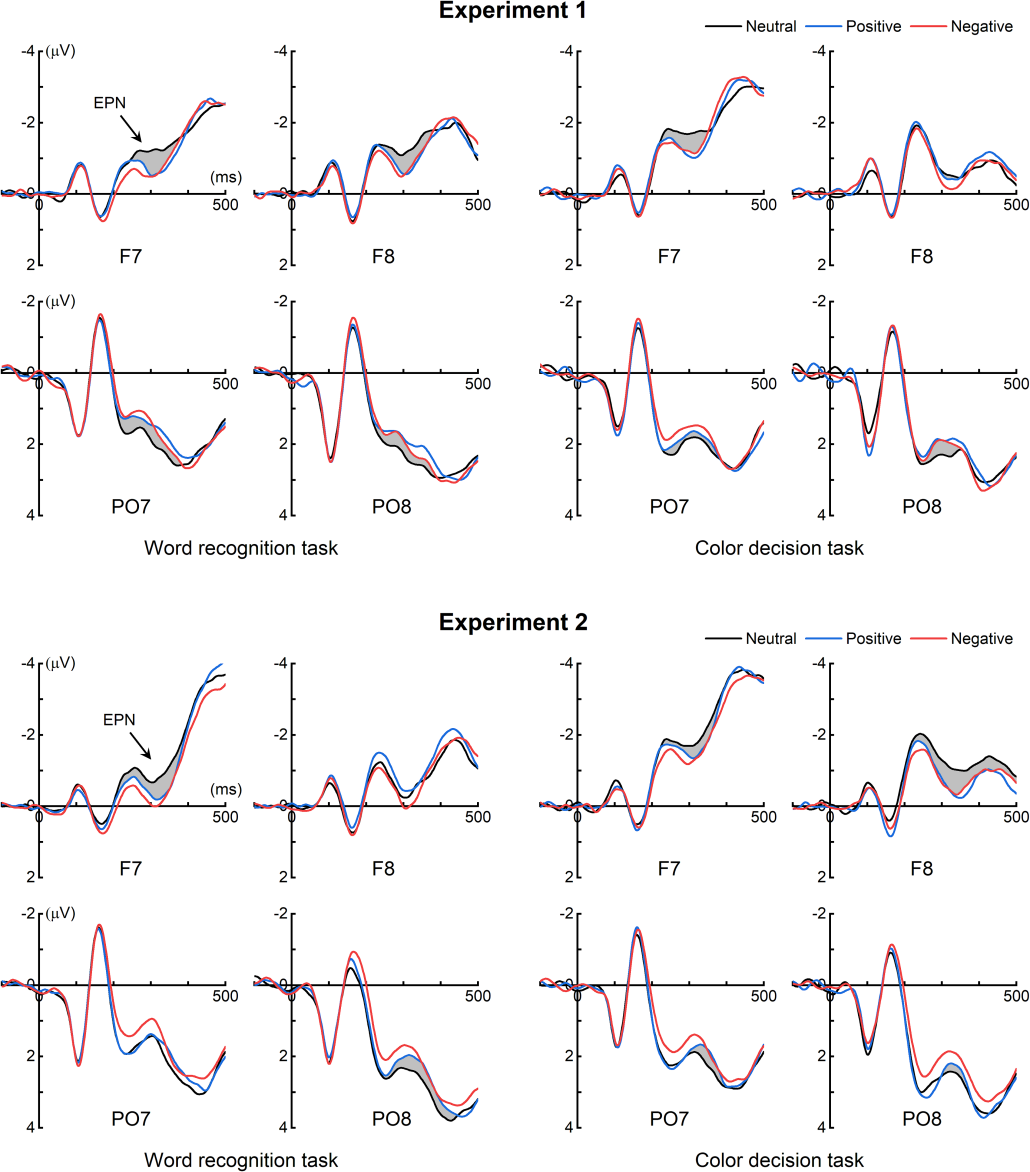
Grand-averaged ERPs. For Experiment 1, in both tasks, emotional words elicited more pronounced negative waves compared to neutral words around 300 ms at the posterior electrode sites, accompanied by enhanced positive waves at the frontal electrode sites. This pattern aligns with the well-established early posterior negativity (EPN) response to emotional stimuli. For Experiment 2, the EPN was evident in the frontal region but less pronounced at the posterior electrode sites.

Figure 2 illustrates the grand-averaged ERPs for each task across both experiments. In Experiment 1, a visual inspection of the ERP waveforms reveals that emotional words, across both tasks, elicited more pronounced negative deflections compared to neutral words at posterior electrode sites (e.g., PO7 and PO8), and more pronounced positive deflections compared to neutral words in the frontal region (e.g., F7 and F8). This observed pattern closely corresponds to the topography of the EPN response to emotional stimuli (e.g., Kissler et al., 2007, 2009; Schacht and Sommer, 2009b; Bayer et al., 2012b; Chen et al., 2015). In contrast, for Experiment 2, the ERP difference between emotional and neutral words was notable in the frontal region (e.g., F7 and F8) but less pronounced at the posterior electrode sites (e.g., PO7 and PO8).

**Figure 3:**
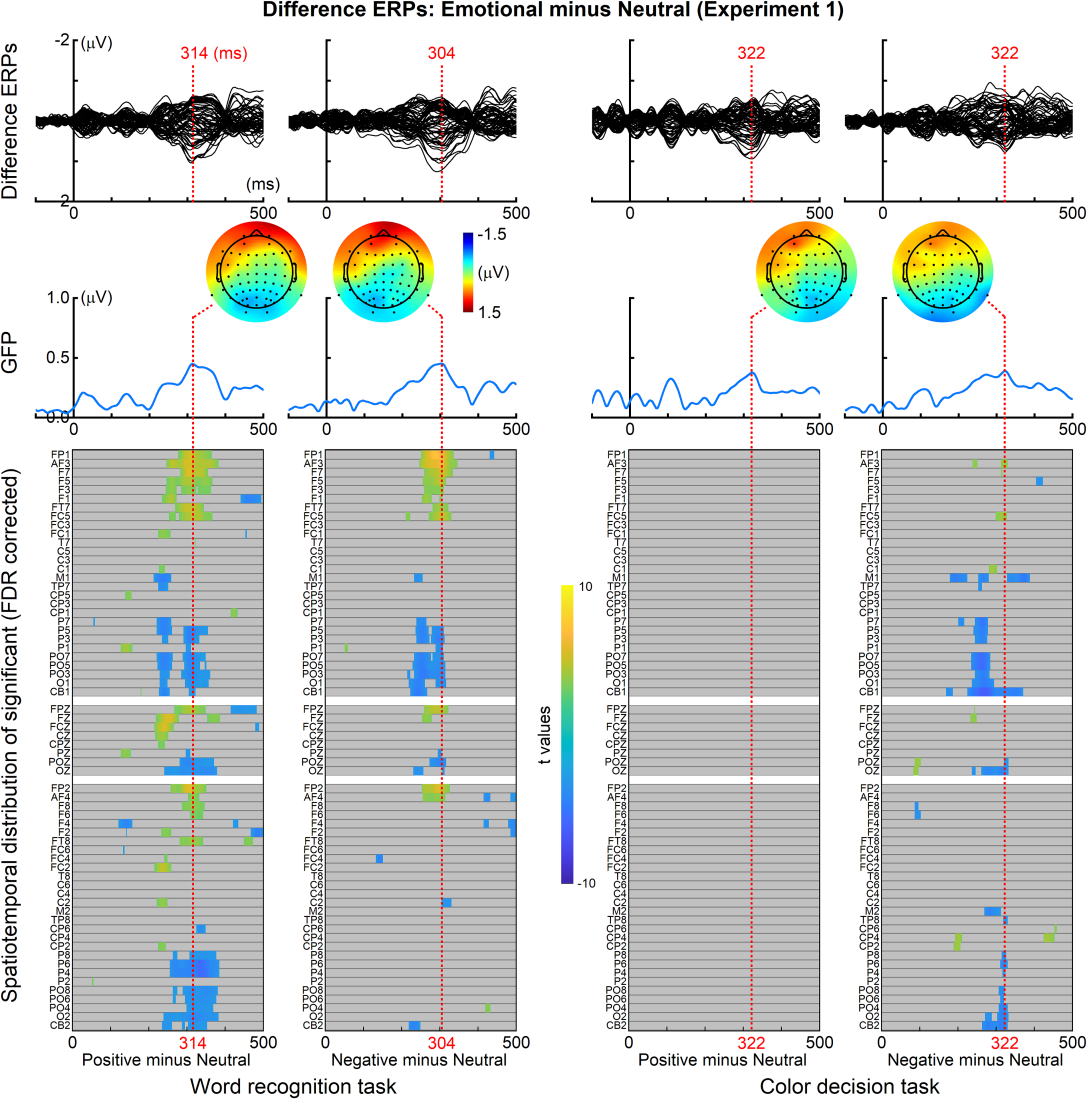
ERP differences between emotional and neutral words (Experiment 1). Top panels: grand-averaged difference ERPs across all 64 recording electrodes, obtained by subtracting ERPs elicited by neutral words from those elicited by emotional words. Middle panels: global field power (GFP) plots representing the computed GFP of the difference ERPs for each task. Bottom panels: spatiotemporal distribution of significant time points resulting from repeated-measures two-tailed *t*-tests conducted on ERPs elicited by emotional and neutral words at each electrode across time points from 50 to 500 ms.

**Figure 4:**
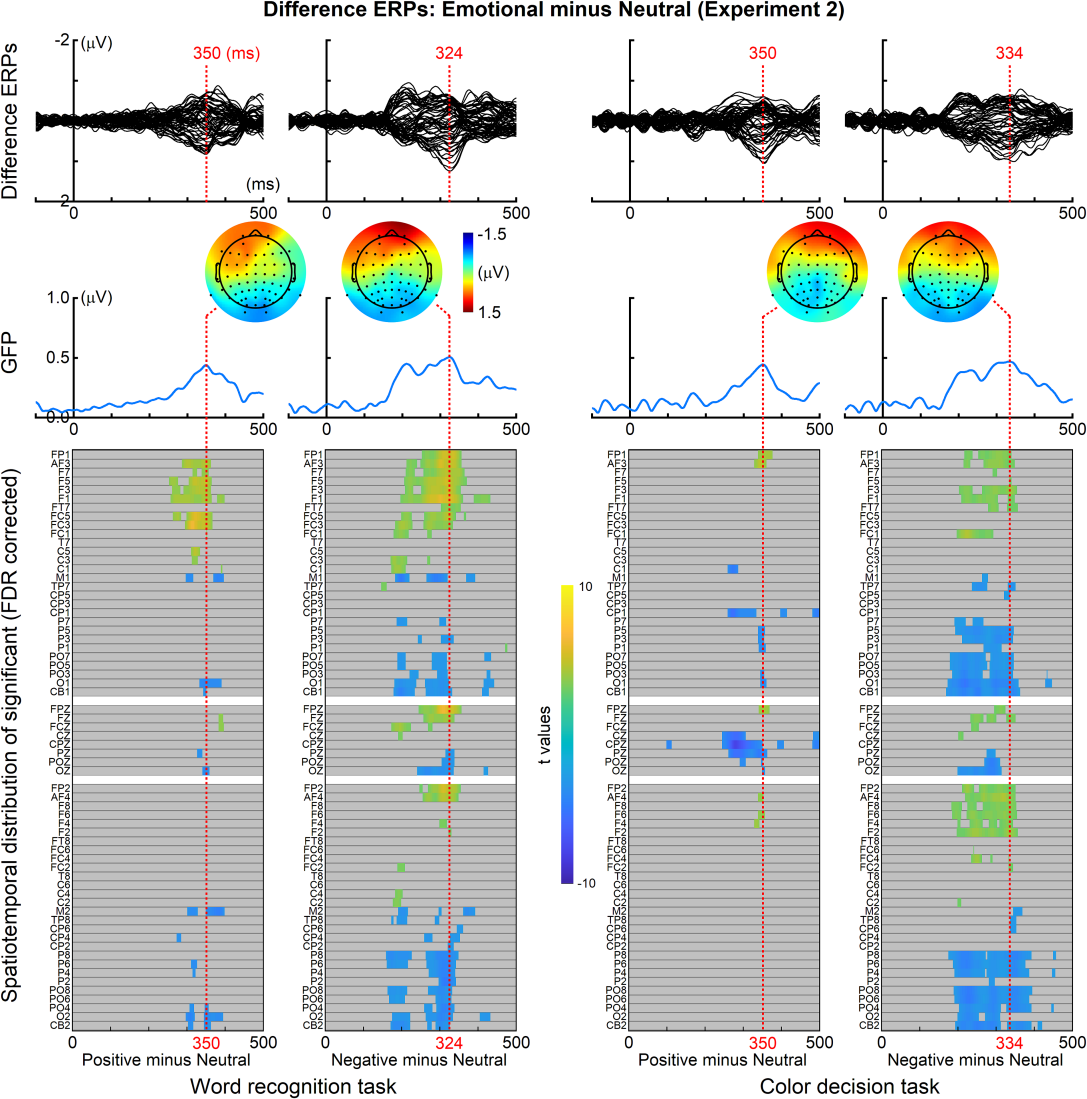
ERP differences between emotional and neutral words (Experiment 2). Top panels: grand-averaged difference ERPs across all 64 recording electrodes, obtained by subtracting ERPs elicited by neutral words from those elicited by emotional words. Middle panels: global field power (GFP) plots representing the computed GFP of the difference ERPs for each task. Bottom panels: spatiotemporal distribution of significant time points resulting from repeated-measures two-tailed *t*-tests conducted on ERPs elicited by emotional and neutral words at each electrode across time points from 50 to 500 ms.

For both experiments, to elucidate the differences in ERPs between emotional and neutral words, the ERPs elicited by neutral words were subtracted from those elicited by emotional words. The resulting grand-averaged difference ERPs across all 64 recording electrodes are displayed in Figures 3, 4 (top panels). Additionally, the global field power (GFP) of these difference ERPs for each task was computed and is depicted in Figures 3, 4 (middle panels). Moreover, for each task, the ERPs elicited by emotional and neutral words at each electrode underwent repeated-measures two-tailed *t*-tests across all time points from 50 to 500 ms. The resulting spatiotemporal distribution of significant time points, corrected for false discovery rate (FDR) to account for multiple comparisons, is presented in Figures 3, 4 (bottom panels).

As depicted in Figure 3 (bottom panels), in Experiment 1, a significant ERP difference was observed around 300 ms between positive and neutral words, as well as between negative and neutral words, in the word recognition task. In contrast, for the color decision task, a significant ERP difference was evident around 300 ms specifically between negative and neutral words. Although no statistically significant difference was detected between the ERPs to positive words and neutral words in the color decision task, a notable difference around 300 ms was apparent in both the ERPs and GFP plots. Moreover, the topographic distribution of the difference ERPs, peaking around 300 ms across all four comparisons, closely mirrors the typical topographic distribution of the EPN response, characterized by positive amplitudes in the frontal region and negative amplitudes in the occipitotemporal area (Kissler et al., 2007, 2009; Schacht and Sommer, 2009b; Bayer et al., 2012b; Chen et al., 2015).

As shown in Figure 4 (bottom panels), in Experiment 2, a significant ERP divergence was evident around 300 ms between positive and neutral words, as well as between negative and neutral words, across both the word recognition and color decision tasks. The topographic distribution of the difference ERPs, peaking around 300 ms for all four comparisons, also aligns with the typical topographic distribution of the EPN response. Notably, the ERP responses to negative words compared to neutral words also showed a statistically significant difference around 220 ms (see bottom panels in Figure 4 and see Supplementary Figure S1 for similar results when comparing negative and positive words). The time course and spatial distribution of the ERP difference around 220 ms align with the ERP signature of accessing lexical orthographic representations (Yu et al., 2022; Huang et al., 2023; Zhang et al., 2023). These results corroborate previous findings on the inhibitory effect of emotional processing on negative words (e.g., Ohman and Mineka, 2001; Estes and Verges, 2008; Grandjean and Scherer, 2008; Scott et al., 2009; Ben-David et al., 2012; Wu and Thierry, 2012; Kuperman et al., 2014). This incidental finding is further discussed in the Supplementary material.

### 3.3 Source analysis results

**Figure 5:**
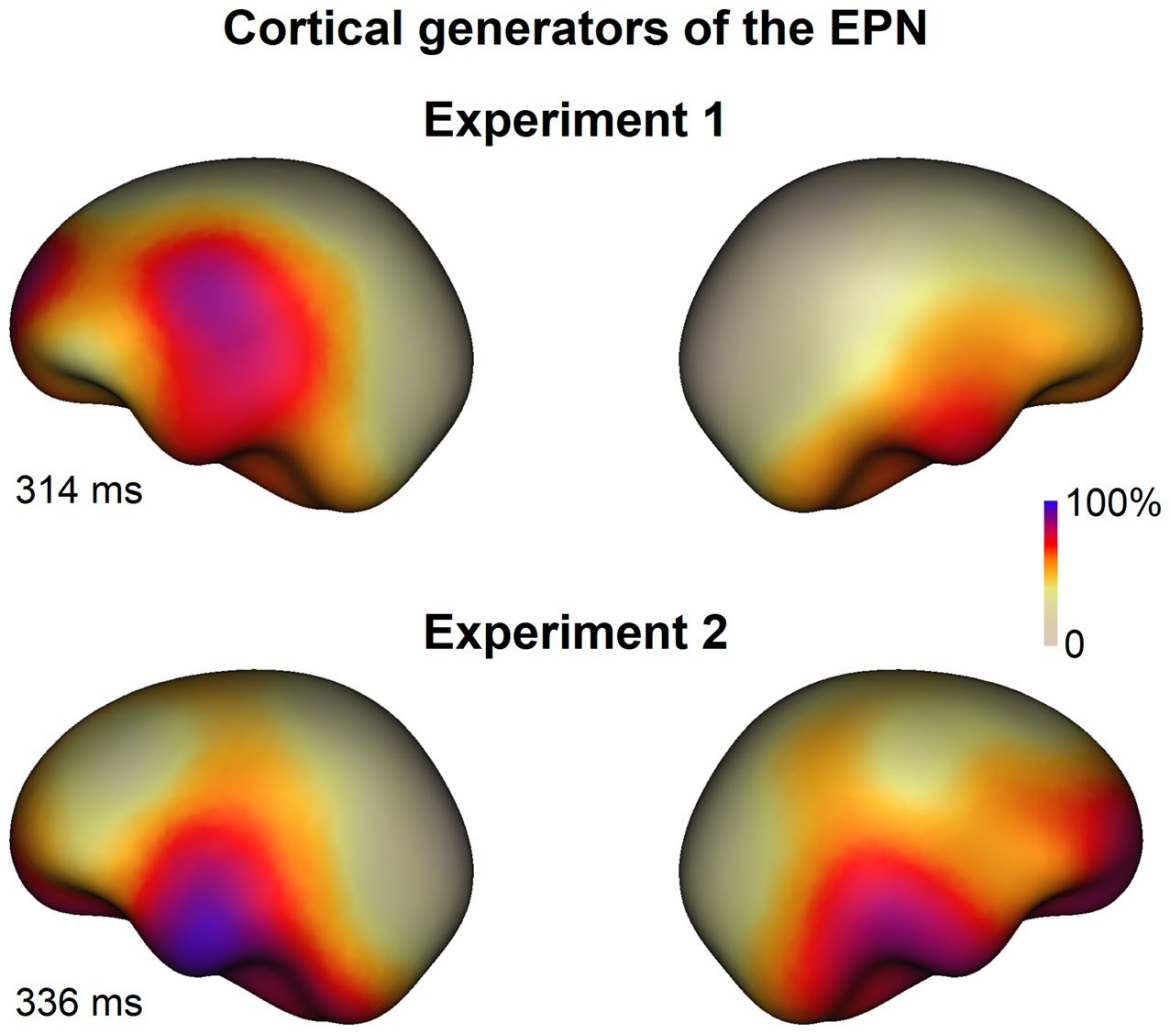
Source analysis results. The left temporal lobe might be the cortical generator of the EPN response for both experiments.

For each experiment, we computed the difference ERPs (i.e., EPN response) between emotional and neutral words across the two tasks. These difference ERPs were then grand-averaged across all participants. Subsequently, we identified the sources of the grand-averaged difference ERP at the peak of the GFP, specifically at 314 ms for Experiment 1 and 336 ms for Experiment 2. The findings revealed the left temporal lobe as the likely cortical generator of the difference ERP for both experiments, as depicted in Figure 5.

## 4 Discussion

Decoding emotional cues from diverse sources like faces, images, and visual words is essential for human interaction. A neural correlate of this emotional processing often manifests as the EPN component of ERP. However, there’s limited evidence of the EPN response to Chinese emotional words in prior studies. Given the unique logographic nature of Chinese script and its lexicon rich in compound words, this study aimed to determine whether EPN could be elicited by Chinese one-character monomorphic words in Experiment 1, and by two-character compound words in Experiment 2. Through a data-driven mass univariate analysis, EPN was consistently identified in both experiments. These findings help fill the gap in the literature, as evidence concerning the EPN response to Chinese emotional words is notably scarce, providing valuable insights into the neural mechanisms underlying Chinese emotional word comprehension.

### 4.1 The EPN response elicited by Chinese emotional words

The current study observed significant differences in brain responses to emotional and neutral stimuli, particularly around 300 ms after word onset, in both Experiment 1 and Experiment 2. We posit that this 300 ms ERP difference represents the EPN component for several reasons: Firstly, the morphology of the difference ERP, including its peak latency (~300 ms) and its topographical distribution (positive amplitude over the frontal scalp area and negative amplitude over the occipitotemporal scalp area) (refer to Figures 3, 4), aligns closely with findings from previous research on the EPN (Kissler et al., 2007, 2009; Schacht and Sommer, 2009b; Chen et al., 2015). Secondly, the observed ERP difference around 300 ms in our study emerged when contrasting emotional stimuli with neutral ones, rather than when comparing positive and negative stimuli. This aligns with established research indicating that the EPN are often observed for positive or negative words relative to neutral ones (e.g., Kissler et al., 2007, 2009; Herbert et al., 2008; Franken et al., 2009; Zhang et al., 2014). Thirdly, the results of our source analysis revealed that the cortical generators of the observed difference ERP in our study are primarily located in the left temporal lobe (see Figure 5). This region is adjacent to the cortical generators of the EPN in the left-hemisphere extrastriate areas, as reported in prior research (Kissler et al., 2007). The slight location discrepancies may stem from the inherent limitations of ERP source analysis methods in pinpointing precise cortical generators. Lastly, the consistent observation of the difference ERP around 300 ms in both experiments, which employed monomorphic and compound words respectively, provides converging evidence that these difference ERPs reflected emotional processing. In short, based on these findings and comparisons with existing literature, we conclude that the ERP difference around 300 ms observed in both Experiment 1 and Experiment 2 is indicative of the EPN component. Thus, the EPN response can be elicited by Chinese emotional words.

The present study used the global average as the reference electrode. In Experiment 1, the EPN was prominent at the occipitotemporal electrode sites, but this was not observed in Experiment 2 (see shaded areas at electrodes PO7 and PO8 in Figure 2). When the ERPs in both experiments were re-referenced to the mean mastoids, the EPN at the occipitotemporal electrode sites was barely detectable in both Experiment 1 and Experiment 2 (refer to Supplementary Figure S2). These findings suggest that the infrequent detection of the EPN response in prior studies likely stems from two factors. Firstly, the utilization of mean mastoids as an offline reference may not effectively reveal the EPN response, given the close proximity of the occipitotemporal electrode sites to the mastoids. Secondly, when Chinese two-character words are utilized as stimuli and the ERP is referenced to the global average, the EPN remains hardly discernible at the occipitotemporal electrode sites (see Figure 2).

The EPN elicited by emotional faces and pictures has been considered indicative of emotional processing (e.g., Junghofer et al., 2001; Schupp et al., 2004a; Kulke, 2019), and its manifestation in implicit tasks (such as the emotional Stroop task) further underscores spontaneous emotional processing. The EPN elicited by words signifies that emotional processing extends to materials with learned emotional significance (Kissler et al., 2007). Additionally, our current findings indicate that emotional processing of visual words, as indexed by the EPN, remains consistent across different writing systems and across both monomorphic and compound words. Therefore, the EPN response could confidently serve as an emotion-related ERP component in future research utilizing both Chinese monomorphic words and compound words as materials. However, careful consideration is warranted when interpreting the EPN response elicited by emotional compound words, as elaborated below.

### 4.2 The EPN response elicited by emotional compound words

As listed in Table 1, many prior studies have delved into the processing of emotional words using Chinese two-character words as stimuli. However, a crucial aspect often overlooked is the inherent compound nature of Chinese two-character words. A wealth of behavioral and neuroimaging investigations has collectively indicated that compound words are processed concurrently both as a whole unit (whole-word processing) and as a combination of their constituent morphemes (decomposing processing) (for an overview, see Huang et al., 2023). The current study unveiled that the EPN is elicited by compound words (i.e., Chinese two-character words in Experiment 2) as well as monomorphic words (i.e., Chinese one-character words in Experiment 1).

Nevertheless, in this study, many constituent morphemes within the two-character words carry emotional connotations consistent with their corresponding compound words, such as “笑” (meaning smiling) in “笑脸” (meaning smiling face), and “病” (meaning sick) in “病毒” (meaning virus). Consequently, it is unable to discern whether the elicitation of the EPN response is attributed to the holistic word meaning, the meanings of constituent morphemes, or both. Notably, certain two-character words possess emotional connotations, yet their constituent morphemes remain neutral (e.g., “表扬,” meaning praise, where “表” means watch, and “扬” means raise). It remains unclear whether the EPN could be elicited using these semantically opaque compound words. In short, further investigation is needed to elucidate whether the EPN is evoked by the meaning (emotional connotation) of the whole word or by the meanings of constituent morphemes.

### 4.3 Exploring Chinese emotional word processing with EPN in future research

As previously mentioned, EPN is a valuable tool in emotion research. This study sets the stage for future investigations using EPN as an emotion-related ERP component to delve into the processing of emotional words in Chinese. For instance, researchers could employ EPN to explore cross-language variations in emotion processing, to investigate the understanding of emotional connotations by Chinese L2 learners, and to explore the atypical processing of Chinese emotional words in specific populations. Additionally, EPN’s sensitivity to word meaning, particularly emotional connotations, could help to elucidate the mechanisms underlying Chinese compound word processing. For instance, if subsequent research demonstrates that EPN is elicited by emotional compound words consisting of neutral constituent morphemes, it would provide compelling evidence for the holistic processing of Chinese compound words.

Building upon the present findings, we suggest that future research optimize the use of monomorphic words (Chinese one-character words) as stimuli, as the EPN elicited by compound words (Chinese two-character words) is scarcely discernible at occipitotemporal electrode sites (see Figure 2). Alternatively, researchers can calculate the GFP waveform to evaluate the EPN (e.g., Schacht and Sommer, 2009b; Bayer et al., 2012b). GFP, a reference-independent measure of response strength, permits a comprehensive assessment of the entire electrode set (Lehmann and Skrandies, 1980; Murray et al., 2008). A GFP peak around 300 ms, along with the topographic map at the peak, plays a pivotal role in identifying the EPN. This is because the EPN typically peaks around 300 ms and demonstrates positive amplitude over the frontal scalp area and negative amplitude over the posterior scalp area when applying the global average as the reference electrode.

## 5 Conclusion

The EPN is an ERP component elicited by emotional connotations from various stimuli, including faces, images, and visual words. It has been extensively utilized as an emotion-related ERP component in numerous studies. The present study reveals that the EPN can be elicited by Chinese emotional words, regardless of whether they are monomorphemic or compound in structure. This suggests that the EPN can reliably serve as an emotion-related ERP component for investigating emotional processing in future studies employing Chinese words as stimuli.

## Data availability statement

The raw data supporting the conclusions of this article will be made available by the authors, without undue reservation.

## Ethics statement

The studies involving humans were approved by Ethics Committee of Sichuan University. The studies were conducted in accordance with the local legislation and institutional requirements. Written informed consent for participation in this study was provided by the participants’ legal guardians/next of kin.

## Author contributions

KZ: Conceptualization, Data curation, Formal analysis, Funding acquisition, Investigation, Methodology, Project administration, Visualization, Writing – original draft. JL: Formal analysis, Investigation, Writing – review & editing. FG: Formal analysis, Methodology, Supervision, Validation, Visualization, Writing – review & editing.

## Footnotes

^1^ https://en.wikipedia.org/wiki/Chinese_language
