# Supplementary material for: Processing of emotional connotations in Chinese monomorphic and compound words reflected by the early posterior negativity
Source: Front Psychol. 2024 Aug 9;15:1426383. doi: 10.3389/fpsyg.2024.1426383 (PMC11342526; doi:10.3389/fpsyg.2024.1426383)
Supplement: Supplementary file 1 [file Data_Sheet_1.PDF]

![frontiers]

Supplementary Material

1 Supplementary Figures

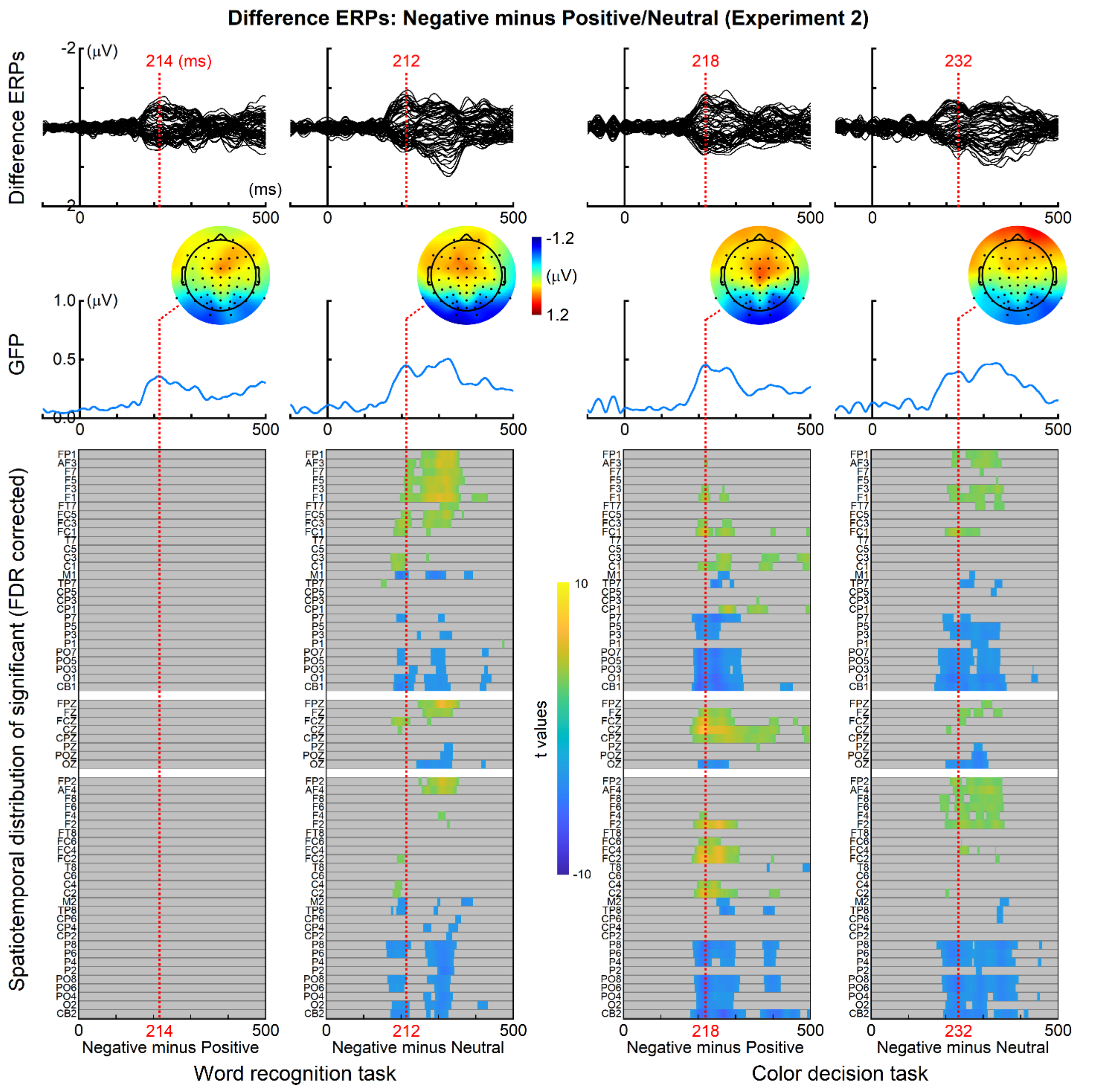

Figure S1. ERP differences between negative and positive/neutral words (Experiment 2). Top panels: Grand-averaged difference ERPs across all 64 recording electrodes, obtained by subtracting ERPs elicited by negative words from those elicited by positive/neutral words. Middle panels: Global field power (GFP) plots representing the computed GFP of the difference ERPs for each task. Bottom panels: Spatiotemporal distribution of significant time points resulting from repeated-measures two-

tailed *t*-tests conducted on ERPs elicited by emotional and neutral words at each electrode across time points from 50 to 500 ms.

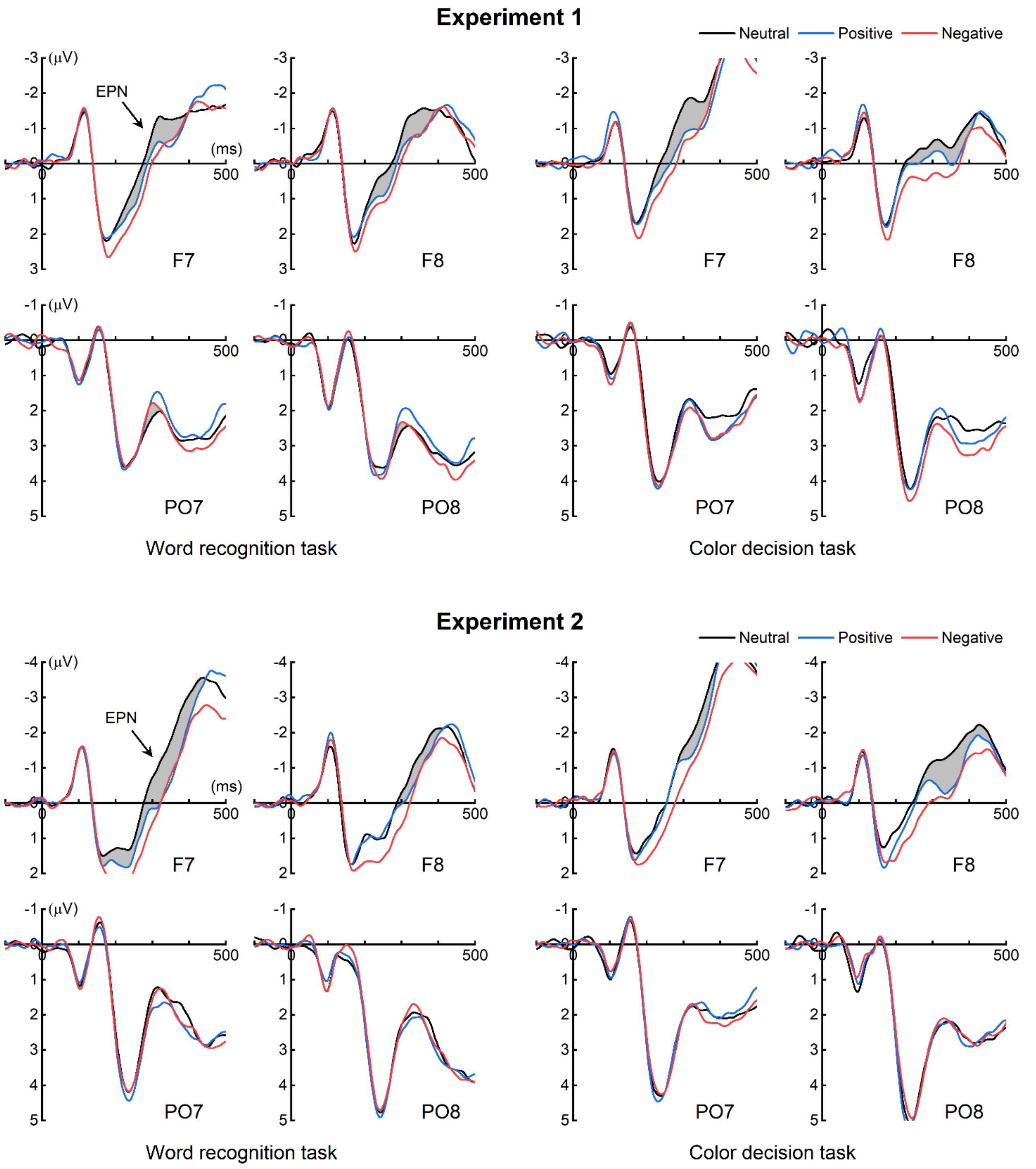

Figure S2. Grand-averaged ERPs (re-reference to mean mastoids). For both experiments, the EPN was evident in the frontal region but scarcely observable at the posterior electrode sites.

2 Supplementary Tables

Known words			Unknown words (Rare words)		
**Positive**	**Negative**	**Neutral**			
真	死	表	芟	肻	郇
笑	离	声	苫	盈	觌
爱	病	院	悫	眚	觔
益	害	城	芴	詈	淯
喜	杀	船	奁	裒	眎
吉	苦	铁	杲	恧	邡
朋	伤	岛	茇	羙	邠
智	痛	闻	罣	蒴	坫
恩	恶	楼	[illegible]	茝	沲
康	危	票	夵	笆	眭
侠	坏	港	[illegible]	耑	邶
幸	罪	普	贳	罝	耖
奖	败	馆	㫒	[illegible]	耴
妙	恨	塔	窆	耋	袉
奋	恐	库	恝	[illegible]	讥
赏	损	售	笇	[illegible]	垤
恋	毒	袋	羑	尜	卸
欣	怨	矿	椠	衰	秠
寿	烂	岩	莛	奭	栝
祝	残	陆	英	郯	轵
吻	悲	室	[illegible]	乩	徂
愉	患	钟	盇	劬	泖
甜	烦	椅	荼	袷	旴
悦	疾	瓶	菑	彤	眵
孝	灾	池	罙	觇	袝
财	贬	峡	坌	绂	舲
欢	丧	铜	莾	砟	贮
善	污	齿	莧	沔	仉
香	骂	岗	眘	铦	祜
宝	腥	座	苅	斛	邦
玩	骗	盐	訾	[illegible]	跕
丽	惨	壳	岽	铕	剞
诚	伪	胃	昃	郤	硁
勇	盗	柴	岙	砢	秬
敬	脏	柜	荅	眍	眹
庆	惩	亭	菉	汔	陙
荣	辱	盆	竒	既	轪
伴	忌	盒	豈	眖	竚
忠	奸	框	覍	阽	庋
净	葬	伞	若	仂	阋
祥	窃	渠	筇	绖	阒
贺	险	砖	罖	訢	尥
俊	淫	饼	穸	埭	逃
帅	讽	巷	哿	紬	屃
夸	哀	桶	荦	贶	迺
旺	坟	壶	芼	驵	达
宠	冤	言	蚩	靰	飑
娱	诈	洞	穵	紤	[illegible]
萌	疮	桨	覂	邲	遆
俏	贱	话	砦	舡	逌

Table S1. Stimuli used in Experiment 1

Known words			Unknown words (Pseudowords)		
Positive	Negative	Neutral			
漂亮	敌人	玻璃	身果	贸饮	闯元
快乐	逼迫	进入	次度	曹幼	圳葡
满意	可怕	脑袋	报才	忍页	仑芝
敬仰	悲痛	报纸	接必	躺钢	懒吁
舒服	愤怒	椅子	治展	奈扣	罗陕
勇气	失望	普通	象双	拨滴	浦讶
关怀	批评	晚饭	张卖	筹丛	脾弓
享受	难过	胳膊	转夫	紫仲	舟吼
礼物	委屈	浴室	位该	遥吕	僚沫
灿烂	忧郁	隐秘	标谈	坛沈	柄坑
良心	病毒	基本	众构	敲纹	芒湘
活泼	棺材	接近	律足	侯抖	筋兆
笑脸	残酷	月饼	斗束	炼恒	厘焰
宝贝	尸体	变化	神各	臣纺	宛咸
庄严	欺骗	摆动	倒旅	弥围	弦坠
崇拜	埋怨	对称	承盖	肚染	谱煌
潇洒	凄凉	店员	盟更	抚亚	溶俯
乖巧	不屑	长期	袄恪	仆郭	烛硕
奇迹	流氓	水缸	掉括	宇乳	松勿
香味	汉奸	散发	编凌	添摩	郡昆
体面	囚犯	抽屉	晋毫	垒蒋	斯甸
信念	损害	编辑	侃咪	挣汪	茂栗
贡献	憔悴	打印	端逸	车讲	拢喏
积极	伤口	奔走	咽泽	朵中	泼咨
亲密	恶心	相同	伦岸	寸扔	枢姚
幽默	痛苦	固定	载洛	丘匹	矣蒲
深情	虚伪	圆形	跳套	苗肠	刮郊
完美	地狱	连绵	练搬	韵笼	爪陡
自豪	轻蔑	弯曲	卷坦	挡纲	晒确
掌声	冤枉	笔直	祖丝	卧膜	咳衔
表扬	害怕	档案	蒙管	荀纳	巩绢
欣赏	堕落	胡须	弄则	窝睁	媳姜
梦想	鸦片	澡盆	替塞	铃填	带沾
向往	下贱	悠长	栈戎	扮杆	曾早
尊严	烦闷	侧面	街促	邵胎	疏咕
福气	混蛋	均匀	阮扎	饱茫	杖绣
丰收	谣言	细密	搞山	挨蓝	坎轿
诚恳	反感	信箱	芭茄	丈乾	钻敦
友谊	晦气	成群	杠吭	咐介	服沼
欢呼	刻薄	潮湿	奉旋	泊肺	京涅
雅致	无耻	短暂	菲杯	斑涛	衬俱
勤快	坟头	证明	绪粗	盒温	昌兜
机智	遗物	橡皮	皆租	玑拆	洪邓
杰作	陷阱	泳池	悄扫	辽抹	桓汁
救星	厄运	化肥	手土	倘冻	佣汝
愿望	狡猾	毛巾	贯侦	驰栏	勒齐
喜讯	瞎话	绳索	皂燕	每却	钓析
忠实	破坏	公共	泥扩	顽颈	麻丐
奖状	墓碑	眼罩	拣牡	收广	倚江
园丁	出殡	垂直	吃巡	尉朴	茎缅

Table S2. Stimuli used in Experiment 2

3 Supplementary Discussion

Inhibitory effect of orthographic processing of Chinese negative compound words

The emotional processing of negative words often appears to manifest an inhibitory effect. For instance, Ben-David et al. (2012) identified an emotional Stroop effect, where individuals exhibited slower response times in naming the ink color of negative emotion-laden words compared to neutral words. Similarly, Estes and Verges (2008) documented that negative words (e.g., "spider") were associated with slower lexical decisions. Additionally, Kuperman et al. (2014) found that greater negativity generally resulted in slower reaction times for lexical decision and naming tasks. This inhibitory effect may be reflected in the modulation of P1 or N170 ERP components (Scott et al., 2009; Bayer et al., 2012; Yao et al., 2016), which could signify the prioritized attraction of attention by negative stimuli (Ohman and Mineka, 2001), an adaptive response to negative stimuli (Grandjean and Scherer, 2008), or a lack of control over variables such as familiarity (for a review see Hinojosa et al., 2020).

Although the lexical decision times to positive, negative, and neutral words were not available in the present study, the ERP results revealed a potential inhibitory effect of emotional processing on negative words. Specifically, in Experiment 2, differences in ERP responses to negative words compared to neutral words were observed not only around 300 ms (i.e., the EPN) but also around 220 ms (see Figure S1). The ERP difference around 220 ms was also observed when comparing negative and positive words (see Figure S1). In contrast, the 220 ms difference was not observed when comparing positive and neutral words (see Figure 4 in the main text). The time course and spatial distribution of the ERP difference around 220 ms align with the ERP signature of accessing lexical orthographic representations of Chinese words (Yu et al., 2022; Huang et al., 2023; Zhang et al., 2023). Therefore, the ERP difference around 220 ms may suggest attenuated lexical orthographic processing of negative words compared to neutral and positive words. These findings seem to support the notion of an inhibitory effect of emotional processing on negative words. However, this inhibitory effect was observed only in Experiment 2, which used compound words, and not in Experiment 1, which used monomorphic words. Thus, the inhibitory effect might reflect inhibited whole-word orthographic processing of compound words. Consistently, Wu and Thierry (2012) found that Chinese compound words with a negative valence are not activated by their English translations as positive and neutral compound words are, which might also result from the inhibitory effect on the orthographic lexical accessing of Chinese negative compound words.

The neural mechanisms underlying the inhibitory effect of emotional processing on negative words remain unclear (for a review see Hinojosa et al., 2020). The present study offers a perspective on the emotional processing of Chinese negative compound words, suggesting that the lexical orthographic representations of Chinese negative compound words may be less robust compared to those of neutral and positive compound words. However, this perspective is speculative and requires further investigation.

4 References

Bayer, M., Sommer, W., and Schacht, A. (2012). P1 and beyond: functional separation of multiple emotion effects in word recognition. *Psychophysiology* 49(7)**,** 959-969. doi: 10.1111/j.1469-8986.2012.01381.x.

Ben-David, B.M., Chajut, E., and Algom, D. (2012). The Pale Shades of Emotion: A Signal Detection Theory Analysis of the Emotional Stroop Task. *Psychology* 03(07)**,** 537-541. doi: 10.4236/psych.2012.37079.

Estes, Z., and Verges, M. (2008). Freeze or flee? Negative stimuli elicit selective responding. *Cognition* 108(2)**,** 557-565. doi: 10.1016/j.cognition.2008.03.003.

Grandjean, D., and Scherer, K.R. (2008). Unpacking the cognitive architecture of emotion processes. *Emotion* 8(3)**,** 341-351. doi: 10.1037/1528-3542.8.3.341.

Hinojosa, J.A., Moreno, E.M., and Ferré, P. (2020). Affective neurolinguistics: towards a framework for reconciling language and emotion. *Language, Cognition and Neuroscience* 35(7)**,** 813-839. doi: 10.1080/23273798.2019.1620957.

Huang, B., Yang, X., Dong, S., and Gu, F. (2023). Visual event-related potentials reveal the early whole-word lexical processing of Chinese two-character words. *Neuropsychologia* 185**,** 108571. doi: 10.1016/j.neuropsychologia.2023.108571.

Kuperman, V., Estes, Z., Brysbaert, M., and Warriner, A.B. (2014). Emotion and language: valence and arousal affect word recognition. *J Exp Psychol Gen* 143(3)**,** 1065-1081. doi: 10.1037/a0035669.

Ohman, A., and Mineka, S. (2001). Fears, phobias, and preparedness: toward an evolved module of fear and fear learning. *Psychol Rev* 108(3)**,** 483-522. doi: 10.1037/0033-295x.108.3.483.

Scott, G.G., O'Donnell, P.J., Leuthold, H., and Sereno, S.C. (2009). Early emotion word processing: evidence from event-related potentials. *Biol Psychol* 80(1)**,** 95-104. doi: 10.1016/j.biopsycho.2008.03.010.

Wu, Y.J., and Thierry, G. (2012). How reading in a second language protects your heart. *J Neurosci* 32(19)**,** 6485-6489. doi: 10.1523/JNEUROSCI.6119-11.2012.

Yao, Z., Yu, D., Wang, L., Zhu, X., Guo, J., and Wang, Z. (2016). Effects of valence and arousal on emotional word processing are modulated by concreteness: Behavioral and ERP evidence from a lexical decision task. *Int J Psychophysiol* 110**,** 231-242. doi: 10.1016/j.ijpsycho.2016.07.499.

Yu, R., Chen, J., Peng, Y., and Gu, F. (2022). Visual event-related potentials reveal the early lexical processing of Chinese characters. *Neuropsychologia* 165**,** 108132. doi: 10.1016/j.neuropsychologia.2021.108132.

Zhang, K., Gu, F., and Yu, H. (2023). Early lexical processing of Chinese one-character words and Mongolian words: A comparative study using event-related potentials. *Front Psychol* 13**,** 1061990. doi: 10.3389/fpsyg.2022.1061990.
